# One-Dimensional ZnO Nanorod Array Grown on Ag Nanowire Mesh/ZnO Composite Seed Layer for H_2_ Gas Sensing and UV Detection Applications

**DOI:** 10.3390/s24175852

**Published:** 2024-09-09

**Authors:** Fang-Hsing Wang, An-Jhe Li, Han-Wen Liu, Tsung-Kuei Kang

**Affiliations:** 1 Graduate Institute of Optoelectronic Engineering, National Chung Hsing University, Taichung 402202, Taiwan; hwliu@dragon.nchu.edu.tw; 2 Department of Electrical Engineering, National Chung Hsing University, Taichung 402202, Taiwan; f5852000@yahoo.com.tw; 3 Department of Electronic Engineering, Feng-Chia University, Taichung 40724, Taiwan; kangtk@fcu.edu.tw

**Keywords:** zinc oxide nanorod, silver nanowire, ultraviolet photodetector, hydrogen gas sensor, hydrothermal

## Abstract

Photodetectors and gas sensors are vital in modern technology, spanning from environmental monitoring to biomedical diagnostics. This paper explores the UV detection and gas sensing properties of a zinc oxide (ZnO) nanorod array (ZNA) grown on silver nanowire mesh (AgNM) using a hydrothermal method. We examined the impact of different zinc acetate precursor concentrations on their properties. Results show the AgNM forms a network with high transparency (79%) and low sheet resistance (7.23 Ω/□). A sol–gel ZnO thin film was coated on this mesh, providing a seed layer with a hexagonal wurtzite structure. Increasing the precursor concentration alters the diameter, length, and area density of ZNAs, affecting their performance. The ZNA-AgNM-based photodetector shows enhanced dark current and photocurrent with increasing precursor concentration, achieving a maximum photoresponsivity of 114 A/W at 374 nm and a detectivity of 6.37 × 10^14^ Jones at 0.05 M zinc acetate. For gas sensing, the resistance of ZNA-AgNM-based sensors decreases with temperature, with the best hydrogen response (2.71) at 300 °C and 0.04 M precursor concentration. These findings highlight the potential of ZNA-AgNM for high-performance UV photodetectors and hydrogen gas sensors, offering an alternative way for the development of future sensing devices with enhanced performance and functionality.

## 1. Introduction

Photodetectors and gas sensors are key to driving modern technology, with applications ranging from optical communications to imaging systems, environmental monitoring, and biomedical diagnostics. Future sensor technologies aim to be thinner, lighter, energy-efficient, and seamlessly integrated with wearable devices [1,2]. Nanostructured materials, especially one-dimensional nanomaterials, occupy an important position in sensor technology due to their unique physical, chemical, and mechanical properties at the nanometer scale, including high aspect ratios and highly anisotropic properties, making them ideal for sensor applications [3,4].

Among the various materials used in photodetection and sensing devices, zinc oxide (ZnO) stands out due to its excellent optoelectronic properties, chemical and thermal stability, non-toxicity, biodegradability, abundance, and relatively low cost, which have garnered significant attention [5]. The unique properties of ZnO nanostructures make them highly suitable for a wide range of applications, including photocatalysts, light-emitting diodes, nanolasers, gas sensors, piezoelectric components, cathode-emitting devices, surface acoustic wave components, optical probes of atomic force microscopes, low-voltage excited fluorescent materials, and photodetectors in the optoelectronics industry [6].

The nanostructured form of ZnO offers distinct advantages such as a high surface-to-volume ratio, high electron mobility, low production costs, tunable properties, and enhanced sensitivity, making it suitable for various sensing applications. Several techniques are available for synthesizing ZnO nanostructures, including metal–organic chemical vapor deposition [7], molecular beam epitaxy, the template-assisted method, pulsed laser deposition [8], chemical bath deposition [9], and the hydrothermal method [10,11]. Among these, the hydrothermal method is particularly advantageous due to its ease of fabrication, scalability to large device areas, physical flexibility, and, most importantly, its low cost. These benefits make it especially suitable for producing flexible semiconductor devices compared to the traditional methods used for fabricating crystalline semiconductor devices [12]. Since ZnO grows at relatively low temperatures, typically around 60–90 °C, it allows for the use of plastic substrates with lower melting points, reducing production costs and enabling the potential development of flexible components. For instance, Aidit et al. demonstrated a flexible temperature sensor with a ZnO nanorods (NRs)/Poly(3,4-ethylenedioxythiophene) polystyrene sulfonate (PEDOT:PSS) composite as an active layer on polyethylene terephthalate (PET) substrates [11]. L. Shi. fabricated silver nanowires (AgNWs) conductive films on PET substrates, achieving a sheet resistance of 13–203 Ω/□ and light transmittance of 89.7–94.6% at 550 nm through a spin-coating method [13]. In the context of gas sensing applications, hydrogen (H_2_) is a clean and renewable energy carrier with the potential to replace fossil fuels. However, due to its high flammability, hydrogen poses a significant risk of fires and explosions, making the development of highly sensitive hydrogen sensors crucial for the advancement of hydrogen as a green energy source. Mohammad et al. reported the fabrication of H_2_ gas sensors using tilted and vertically aligned ZnO NRs on thin nylon substrates via hydrothermal technology [14]. They observed an increase in H_2_ sensitivity from 109 to 264% under 500 ppm H_2_ exposure at temperatures ranging from room temperature to 180 °C. In 2024, Nguyen et al. reported ZnO NRs decorated with Ag nanoparticles on a PI substrate, achieving a visible (λ = 395 nm) responsivity of 40.2 mA/W [15].

By comprehensively examining the advancements and potential of ZnO nanostructured sensors, this study aims to further enhance the properties of ZnO nanostructured sensors. The research focuses on using a silver nanowire mesh (AgNM) to construct a ZnO seed layer and NR sensing array via spin-coating and hydrothermal methods. Specifically, the study explores effects of synthesis solution concentration on the structural, optical, ultraviolet (UV) light detection, and H_2_ gas sensing properties of nanostructured ZnO-based sensors.

## 2. Materials and Methods

### 2.1. Formation of the Silver Nanowire Mesh

Eagle XG glass provided by Corning Co. (Taichung, Taiwan) was used as the substrate, each measuring 2.5 cm × 2.5 cm. The glass substrate was 0.7 mm thick. All chemicals used in this work were analytical grade and applied without further purification. Prior to the formation of the AgNM, the substrates were cleaned using a sequence of ultrasonic baths. The substrates were immersed sequentially in acetone, deionized water, and isopropyl alcohol (IPA), with each step performed in an ultrasonic cleaning machine (Delta DC150, New Taipei, Taiwan) at a frequency of 40 kHz and an output power of 150 W for 10 min. Following the ultrasonic cleaning, the substrates were blown dry with nitrogen and then placed on a hot plate at 90 °C for 10 min. AgNWs with a diameter of 55–75 nm, a length of 10–20 μm, and a concentration of 1 wt% dispersed in IPA were obtained from Well Being Enterprise Co., Ltd. (Taipei, Taiwan). The AgNM was deposited onto the substrates using a two-step spin-coating process. The first step involved spinning at 400 rpm for 15 s, followed by a second spin at 3500 rpm for 30 s. After coating, the substrates were placed on a hot plate at 200 °C and baked for 3 h to ensure the complete evaporation of the IPA from the AgNM layer.

### 2.2. Formation of the Seed Layer

Before growing ZnO NRs, a ZnO seed layer was prepared using the sol–gel method. This method involved dissolving metal salts in alcohol solvents, allowing zinc ions to undergo hydrolysis, polymerization, and aging to form a clear and transparent sol. The steps for preparing the ZnO sol–gel were as follows:

1. Solution Preparation: A total of 6.5844 g of zinc acetate dihydrate was added to a beaker containing 38.2 mL of monoethanolamine (MEA, HOCH_2_CH_2_NH_2_) and 1.8 mL of ethylene glycol monomethyl ether (EGME, CH_3_OCH_2_CH_2_OH), resulting in a 0.75 M Zn^2+^ ion solution.
2. Heating and Stirring: The mixture was heated and stirred at 60 °C for 2 h, then aged in an auto dry box at room temperature for 48 h to obtain a uniform and transparent ZnO gel solution.
3. Spin Coating: The ZnO sol–gel solution was spin coated onto the substrates prepared in Section 2.1 at a rotation speed of 2000 rpm for 30 s.
4. Baking and Cooling: The coated substrates were placed on a hot plate at 300 °C for 10 min and then allowed to cool for 10 min. The spin-coating, baking, and cooling steps were repeated once more to complete the ZnO seed layer formation.

### 2.3. Synthesis of the ZnO Nanorod Array

**Figure 1:**
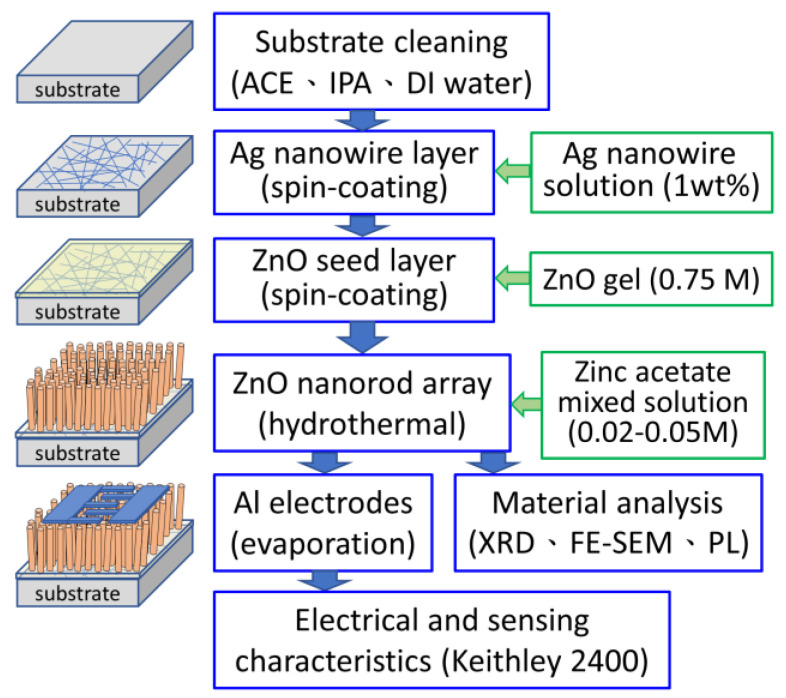
Experimental procedure flow chart and schematic of ZNA-AgNM-based MSM devices.

The ZnO nanorod array (ZNA) was grown using the hydrothermal method. Zinc acetate solutions with concentrations of 0.02 M, 0.03 M, 0.04 M, and 0.05 M were prepared. Hexamethylenetetramine ((CH_2_)_6_N_4_) was added to each solution at the same concentration to act as a conditioner. The substrates prepared in Section 2.2 were immersed in these solutions. The hydrothermal growth process was conducted at 90 °C for 45 min. After the growth process, the substrates were removed from the solution and rinsed with deionized water to eliminate any residual material. The substrates were then baked at 90 °C for 15 min to remove any remaining moisture, completing the growth of the ZNA on the seed layer with the embedded silver nanowire mesh (labeled as ZNA-AgNM). Figure 1 illustrates the experimental procedure flow chart and the schematic of the developed ZNA-AgNM metal–semiconductor–metal (MSM) devices.

### 2.4. Characterization 

#### 2.4.1. Structural and Morphological Characterization

The structural and crystallographic properties of the ZnO nanorod array (ZNA) and the seed layer were characterized using X-ray diffraction (XRD) with Cu-Kα radiation (λ = 1.54056 Å) in a θ–2θ scanning mode (Malvern Panalytical, X’Pert Pro MRD, United Kingdom and The Netherlands). The surface morphology and thickness of ZnO NRs were examined using field emission scanning electron microscopy (FE-SEM, JEOL, JSM-6700F, Tokyo, Japan). The average diameter and length of the nanorods were determined by selecting five nanorods at random from the SEM images. Additionally, the aspect ratio, total surface area, total volume, and surface-area-to-volume ratio of the nanorods were calculated using the following formulas:

aspect ratio = length/diameter(1)

total surface area = length × diameter × π + (diameter/2)^2^ × π × density (2)

total volume = (diameter/2)^2^ × π × length × density (3)

surface-area-to-volume ratio = total surface area/total volume(4)

#### 2.4.2. Optical Characterization

Photoluminescence (PL) is a technique that provides information about energy band gaps, impurities, and defects information. Luminescence properties were analyzed using room-temperature PL spectroscopy (Horiba, iHR550, Kyoto, Japan) with a 325 nm He-Cd laser. The transmission spectrum of the sample was measured using a UV–visible spectrophotometer (Thermo Scientific, Evolution 220, Waltham, MA, USA).

#### 2.4.3. Device Fabrication and Electrical Measurements

Finally, Al interdigital electrodes with a thickness of 200 nm and a spacing of 200 μm were deposited on the ZNA-AgNM structure using thermal evaporation to form an MSM device. Prior to gas sensing measurements, the devices were pretreated by passing dry air through the measurement chamber while heating to 300 °C for 1 h to remove moisture and stabilize the electrical properties. The resistance of the ZNA-AgNM devices was measured at temperatures ranging from 50 °C to 300 °C under varying H_2_ concentrations using a computer-controlled Keithley 2400 source meter (Cleveland, OH, USA). Hydrogen concentration was adjusted by varying the hydrogen-to-dry-air ratio using mass flow controllers. For measurements of photodetection properties, the dark current and photocurrent of the devices were measured using the same Keithley 2400 source meter without or with illumination from a xenon lamp (SOFN, 7ILX150A-UVC, Beijing, China) and a monochromator (SOFN, 7IMU1021, Beijing, China) in a room-temperature auto dry box. The spectral range of the xenon lamp was 0.2−2.5 μm with a power output of 150 W and a luminous intensity of 240 cd.

## 3. Results and Discussion

### 3.1. ZnO Seed Layer Coated on Silver Nanowire Mesh

**Figure 2:**
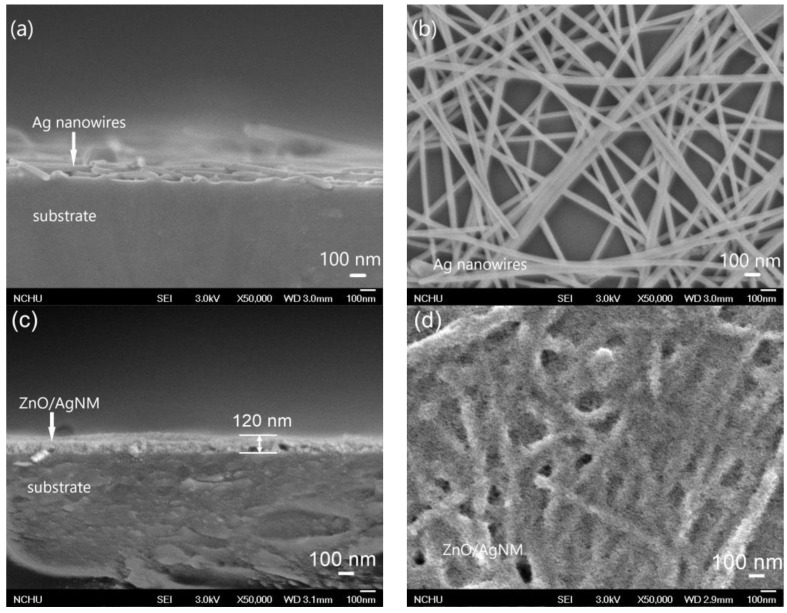
Cross-section view and plane-view FE-SEM images of (**a**,**b**) the silver nanowire mesh (AgNM) and (**c**,**d**) the ZnO seed layer coated on AgNM.

FE-SEM was employed to examine the microstructure of the fabricated materials. Figure 2 presents the cross-sectional and planar SEM views of the spin-coated AgNM and the ZnO seed layer. As shown in Figure 2a,b, the thickness of the AgNM layer was about 105 nm. The SEM images reveal numerous slender, linear AgNWs distributed across the substrate, forming a densely cross-connected mesh structure. The sheet resistance of the AgNM film was measured at 7.23 Ω/□, with an average transmittance of 79% in the visible light range (400−800 nm). These properties demonstrate that the AgNM layer serves as an excellent transparent conductive material, with lower sheet resistance and transmittance compared to previous studies [13], attributed to the denser AgNW network fabricated in this work. Figure 2c,d show that the sol–gel-derived ZnO layer completely encapsulated the AgNM layer. The overall thickness of the ZnO-AgNM composite seed layer was approximately 120 nm.

**Figure 3:**
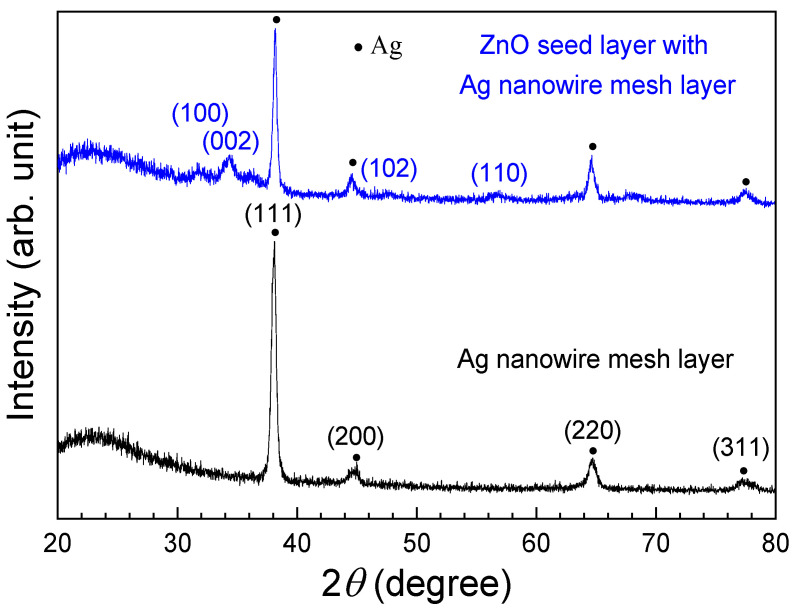
XRD patterns of the AgNM and the ZnO seed layer.

Figure 3 depicts the XRD patterns of the AgNM and the ZnO-AgNM composite seed layer. Both XRD patterns exhibited broadening at 2θ = 20–25°, which could be attributed to the small crystallite size of the silver nanomaterial or strain within the crystal lattice. Similar broadening of the XRD peak in this range for silver nanoparticles was also reported by Anandalakshmi et al. [16]. The AgNM exhibited four distinct diffraction peaks, corresponding to the (100), (111), (200), (220), and (311) planes of face-centered cubic crystals, consistent with the JCPDS card No. 04-0783 [17]. In addition, the ZnO-AgNM composite seed layer, grown using the sol–gel spin-coating method, displayed three ZnO diffraction peaks at 2θ values of 34.62°, 47.64°, and 56.78°, which correspond to the (002), (102), and (110) crystal planes, respectively. These findings align with JCPDS No. 36-1451 [18], confirming that the ZnO seed layer possesses a hexagonal closest-packed wurtzite structure with a preferred orientation along the (002) direction.

### 3.2. ZnO Nanorod Array

**Figure 4:**
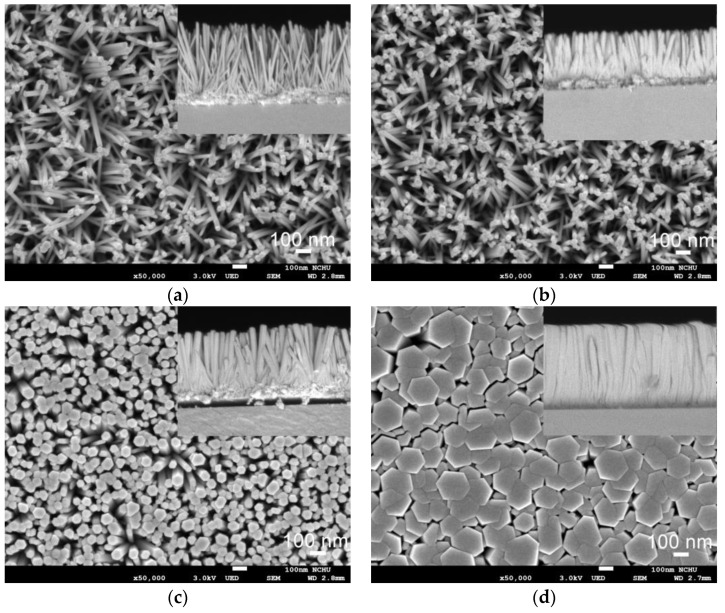
Plane-view FE-SEM images of the ZNAs with different precursor concentrations: (**a**) 0.02 M, (**b**) 0.03 M, (**c**) 0.04 M, and (**d**) 0.05 M. The inset shows their cross-section view.

Figure 4 presents plane-view SEM images of ZNA-AgNM with varying zinc acetate concentrations, with the inset showing the cross-sectional view. Regardless of the concentration, the ZnO NRs exhibited a well-aligned hexagonal pillar shape perpendicular to the substrate surface. The growth mechanism of ZNA-AgNM via the hydrothermal method has been previously discussed in the earlier literature [19,20]. As observed in Figure 4a,b, a lower precursor concentration resulted in thinner and sparser nanorods. When the zinc acetate concentration increased slightly from 0.02 M to 0.03 M, the nanorods diameter increased while their length decreased. This occurs because, at lower concentrations, the increased polar surface area of the (001) plane demands more precursor ions for axial growth. In the absence of sufficient precursor supply, the axial growth rate slows down, leading to shorter nanorods [19,21]. The number of ZnO NRs also slightly increased at this concentration. The inset in Figure 4 shows that the thickness of the ZNA sensing layer approximately matched the average length of the nanorods. The lengths of the nanorods synthesized at zinc acetate concentrations of 0.02 M, 0.03 M, 0.04 M, and 0.05 M were approximately 764 ± 20.3 nm, 515 ± 13.7 nm, 660 ± 15.1 nm, and 884 ± 12.4 nm, respectively, with average diameters of 37 ± 2.2 nm, 49 ± 3.3 nm, 80 ± 6.4 nm, and 226 ± 9.1 nm, respectively. The statistical errors are reported as standard deviations. The total sensing film comprises both the ZNA layer and the ZnO-AgNM seed layer, with the latter having a thickness of approximately 120 nm (as shown in Figure 2). Thus, the overall thicknesses of the sensing films were approximately 884 nm, 635 nm, 780 nm, and 1004 nm for zinc acetate concentrations of 0.02 M, 0.03 M, 0.04 M, and 0.05 M, respectively. As the precursor concentration increased, both the diameter and length of the NRs grew significantly due to a sufficient supply of precursor ions and a strong driving force towards the polar surface of the ZnO seed [19]. However, the area density of the ZNA-AgNM, defined as the number of nanorods per unit area (1 μm^2^), decreased markedly from around 250 to approximately 50 as the zinc acetate concentration increased from 0.02 M to 0.05 M. This inverse relationship between diameter and area density occurs because the number of nanorods that can grow on a given substrate area is limited by their increasing diameter.

**Figure 5:**
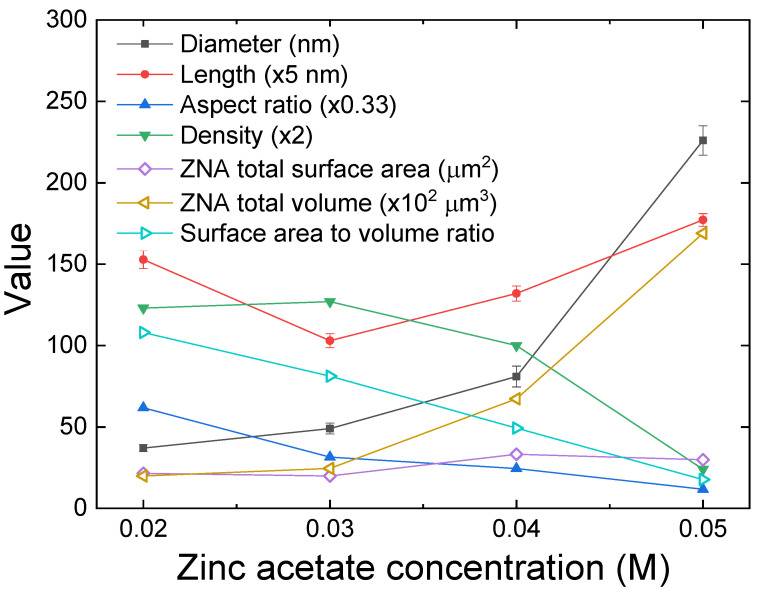
Dimensional parameters of ZNAs with different precursor concentrations.

In addition, the aspect ratio, total surface area, total volume, and surface-area-to-volume ratio of the nanorods were calculated and are displayed in Figure 5. The total surface area and total volume of nanorods were calculated based on a specific substrate area of 1 μm^2^. As the zinc acetate concentration increased from 0.02 M to 0.05 M, the total surface area of the nanorods initially decreased and then increased, with higher values (around 31 μm^2^) observed at concentrations of 0.04 M and 0.05 M, which is advantageous for gas sensitivity. The total volume of the nanorods increased from 0.17 to 1.42 μm^3^ as the zinc acetate concentration rose. Consequently, the surface-area-to-volume ratio decreased from 108 to 18 with increasing zinc acetate concentration. In summary, lower zinc acetate concentrations resulted in higher nanorod density, aspect ratio, and surface-area-to-volume ratio, whereas higher concentrations produced nanorods with higher nanorod length, diameter, total surface area, and total volume. These observations are consistent with similar findings reported in the literature [22] for the ZNA grown on the foamed nickel/ZnO seed layer deposited by sputtering.

**Figure 6:**
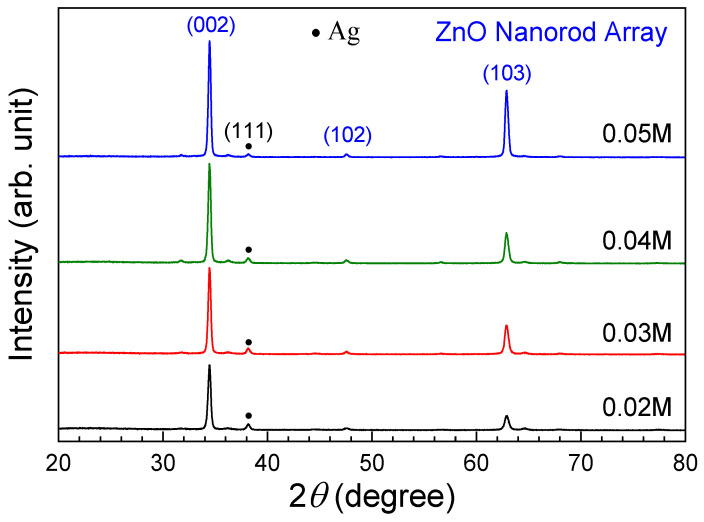
XRD patterns of the ZNA-AgNMs grown with different precursor concentrations.

Figure 6 shows XRD patterns of the ZNA-AgNMs grown with varying precursor concentrations. The diffraction peak at approximately 34.6° in the (002) plane exhibited a significant increase in intensity, indicating a strong preferential orientation of the ZnO NRs along the c-axis. Notably, no characteristic peaks corresponding to silver oxide were detected. The observed XRD peaks aligned well with the standard data from JCPDS No. 36-1451 and ICDD No. 98-002-9272, confirming the hexagonal wurtzite structure of ZNAs [18,23]. As the precursor concentration increased, the intensity of the XRD peaks became more pronounced, which can be attributed to the greater overall volume of the ZNA. This observation is consistent with the trends seen in the SEM images. The average crystallite size of ZnO was calculated using Debye–Scherrer’s equation:

D =  K × λ/(β × cosθ),(5)

where K is the shape factor (0.9), λ is the X-ray wavelength (1.54056 Å), β is the line broadening at FWHM (in radians) of the diffraction peak, and θ is the Bragg angle of the (002) peak [24,25]. It is important to note that Debye–Scherrer’s equation is typically used for determining the size of spherical nanoparticle crystals. In this study, we applied the equation to provide preliminary estimates and insights into the crystallite size and crystal quality of ZnO NRs, despite their elongated shape. As the precursor concentration increased from 0.02 to 0.05 M, the crystallite size correspondingly grew from 26.8 nm to 30.7 nm.

**Figure 7:**
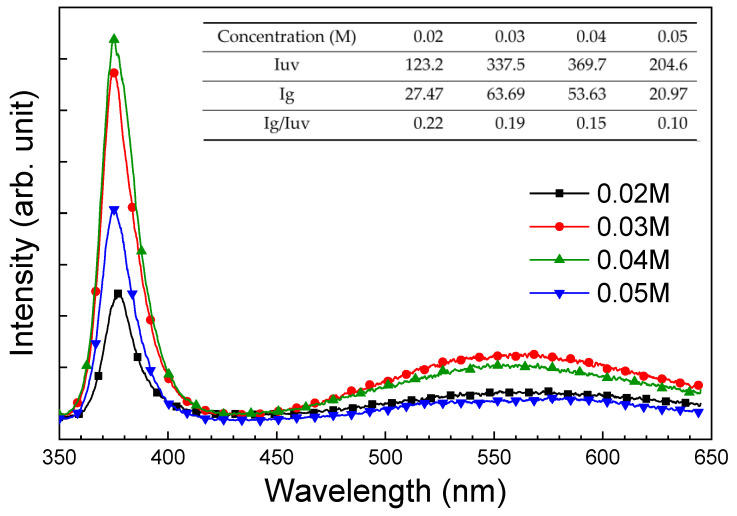
Room-temperature photoluminescence (PL) spectra of ZNA-AgNMs grown with different zinc acetate concentrations.

Figure 7 displays the room-temperature photoluminescence (PL) spectra of ZNA-AgNMs grown with different concentrations of zinc acetate solution. PL analysis provides insights into how changes in growth concentration influence the crystal defects of ZnO. The spectra reveal two prominent emission peaks, a sharp UV emission at around 376 nm (3.30 eV) and a broad green emission band centered at approximately 571 nm (2.17 eV), extending from 460 to 640 nm. The UV emission, also known as near-band edge emission, originates from the recombination of free excitons inherent to ZnO. The source of the green emission remains debated, but it is generally linked to various intrinsic deep-level defects in ZnO, such as oxygen vacancies, zinc vacancies, interstitial zinc, interstitial oxygen, and absorbed hydroxyl group [26]. Lai reported that emissions at 410 nm, 440 nm, 530 nm, and 565 nm in the PL spectrum of ZnO nanomaterials correspond to zinc vacancy, interstitial zinc, oxygen vacancy, and interstitial oxygen, respectively [27]. Qiu et al. identified the yellow luminescence (1.5−2.5 eV) in the PL analysis of well-aligned ultralong ZNAs as originating from absorbed hydroxyl group [28]. In this work, the green emission band between 460 and 640 nm is likely due to oxygen vacancy, interstitial oxygen, and/or absorbed hydroxyl group. The intensity of the green emission was lower than that of the UV emission, indicating a lower concentration of defects.

To more accurately compare nanorod defects and impurities across different samples, the I_defect_/I_UV_ ratio (ratio of defect-level intensity to UV-related luminescence) was calculated. The inset in Figure 7 illustrates the UV and green light emission intensities and their ratio (I_g_/I_UV_) for different zinc acetate solution concentrations. It was observed that I_g_/I_UV_ decreased with increasing zinc acetate concentration, suggesting improved crystal quality and fewer defects at higher precursor concentrations. Previous studies have shown that structural defects significantly influence the electrical properties of ZnO materials, as well as their gas sensing and photodetection performance [29].

### 3.3. Gas Sensing Properties of ZNA-AgNMs

**Figure 8:**
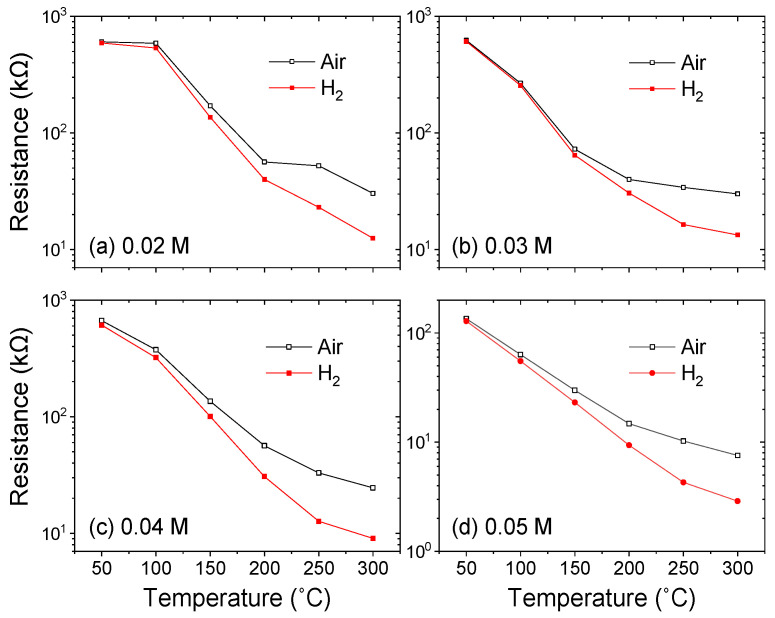
Resistance of the ZNA-AgNM-based gas sensors as a function of operating temperature.

It is well known that sensor response is closely related to operating temperature. To assess the hydrogen gas sensing properties of ZNA-AgNM, the resistance of MSM devices fabricated with ZNA-AgNM was measured at different operating temperatures. The device was placed in a sealed stainless steel chamber, and mass flow controllers were used to regulate the concentration of the test gas. Figure 8 shows the resistance of MSM devices based on ZNA-AgNM grown with varying zinc acetate concentrations, measured in both air and hydrogen environments under 2000 ppm, at temperatures ranging from 50 to 300 °C. Regardless of the concentration of zinc acetate, the resistance of the MSM devices decreased significantly as the operating temperature increased, with the resistance in hydrogen being consistently lower than in air. When the sensor was exposed to air, oxygen molecules were adsorbed onto the ZnO surface, reacting with free electrons from the ZnO NRs to form chemically adsorbed oxygen ions (O_2_^−^(ad) and/or O^−^(ad)) and an electron depletion layer. These reactions can be described by the following equations [30,31,32]:

O_2_(g) → O_2_(ad), (6)

O_2_(ad) + e^−^ (free) → O_2_^−^(ad) (at low temperature), (7)

O_2_^−^(ad) + e^−^ (free) → 2O^−^(ad) (at high temperature), (8)

O^−^(ad) + e^−^ (free) → O^2−^(ad) (at high temperature). (9)

Oxygen molecules and ions capture free electrons from the ZnO conduction band, leading to a reduction in carrier concentration and an increase in ZnO resistance [33]. Upon exposure to hydrogen, a reduction reaction occurs between hydrogen and the chemically adsorbed oxygen ions on the ZnO NRs’ surface, releasing electrons into the conduction band and increasing the current flowing through the MSM device. This reaction is exothermic in nature, rapidly desorbing molecular water from the surface. The reactions are described by the following formulas [34,35]:

2H_2_ + O_2_^−^ (ad) → 2H_2_O + 2e^−^, (10)

H_2_ + O^−^ (ad) → H_2_O + e^−^. (11)

**Figure 9:**
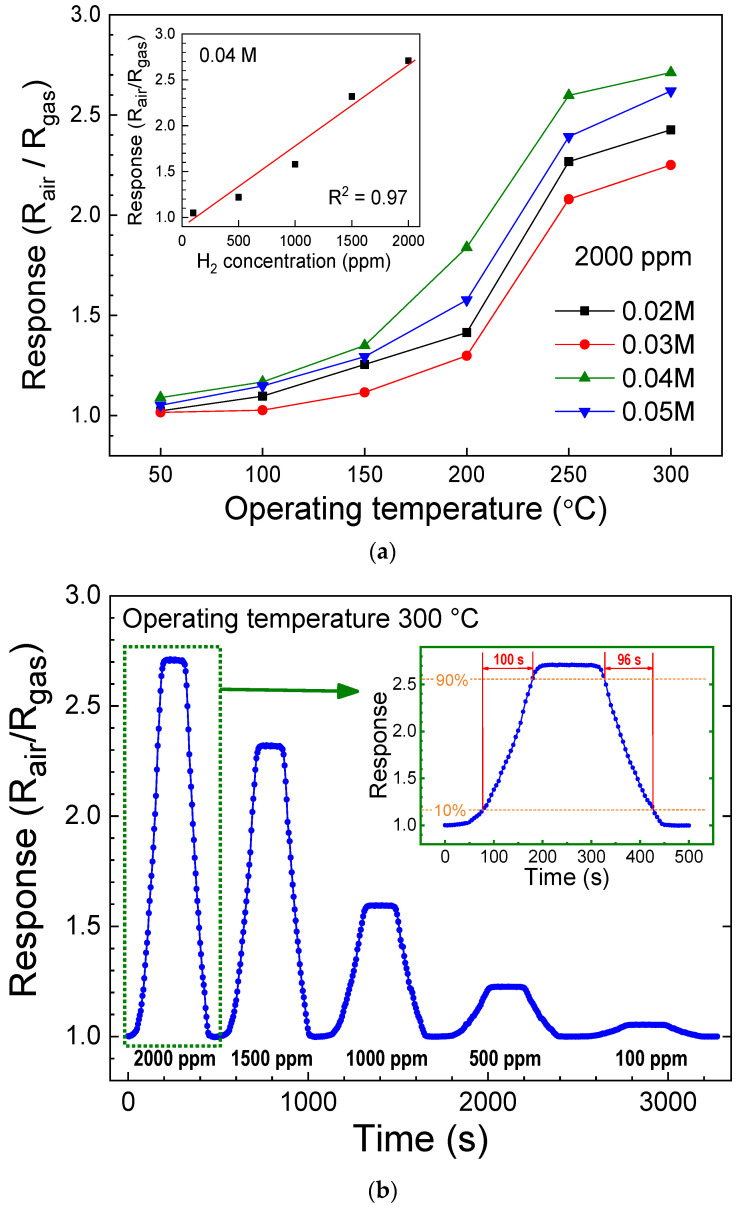
(**a**) H_2_ gas response of ZNA-AgNM-based devices at different zinc acetate concentrations. (**b**) H_2_ response curves of ZNA-AgNM-based devices with a zinc acetate concentration of 0.04 M at 300 °C. The inset highlights the response curve at 2000 ppm H_2_.

To evaluate the performance of gas sensors, the gas response was calculated as the ratio of the sensor’s resistance in synthetic air (R_air_) to its resistance in the analyte gas (R_gas_). Figure 9a shows the H_2_ gas response of the ZNA-AgNM-based devices as a function of operating temperature for different zinc acetate concentrations. The response increased with operating temperature across all zinc acetate concentrations, reaching a maximum at 300 °C. Among the four concentrations tested, the ZNA-AgNM grown at 0.04 M exhibited the highest hydrogen response, with a value of 2.71. This enhanced response is attributed to the ZNA formed at 0.04 M, which possesses a larger total surface area on the seed layer (as shown in Figure 4 and Figure 5). The increased surface area enhances the interaction between the nanorod surface and the analyte gas, allowing more H_2_ molecules to react with the oxygen ions adsorbed on the nanorod surface. This reaction releases electrons back into the ZNAs, increasing the number of free carriers and reducing the resistance.

Previous studies have suggested that the performance of gas sensors is closely linked to the surface-to-volume ratio [34,36,37]. For example, Lupan et al. found that a single ZnO nanowire with a smaller diameter exhibited a higher gas response [34]. However, in this study, we found that the response of the gas sensor is primarily determined by the total surface area of the sensing nanomaterial rather than the surface-to-volume ratio. FE-SEM observations and calculations revealed that ZNAs grown at higher zinc acetate concentrations have a smaller surface-to-volume ratio. The inset of Figure 9a shows the response of the device as the H_2_ concentration varies from 100 to 2000 ppm. The response was approximately linear with gas concentration, with an R-square value of 0.97, indicating an excellent linear relationship between the H_2_ response and gas concentration.

Figure 9b presents the H_2_ response curve of the ZNA-AgNM-based devices at a zinc acetate concentration of 0.04 M at 300 °C, with the inset providing an enlarged view of the response curve at 2000 ppm H_2_. The response and recovery times of the H_2_ sensor were 100 s and 96 s, respectively. Additionally, Table 1 compares the performance of the gas sensor from this study with several ZnO-based hydrogen sensors reported in the literature. These comparisons indicate that decorating ZnO with metal nanoparticles or catalysts is an effective strategy for further enhancing hydrogen sensing properties. Furthermore, reducing the operating temperature of the sensor remains a key area for future development.

### 3.4. Photodetecting Properties of ZNA-AgNMs 

**Figure 10:**
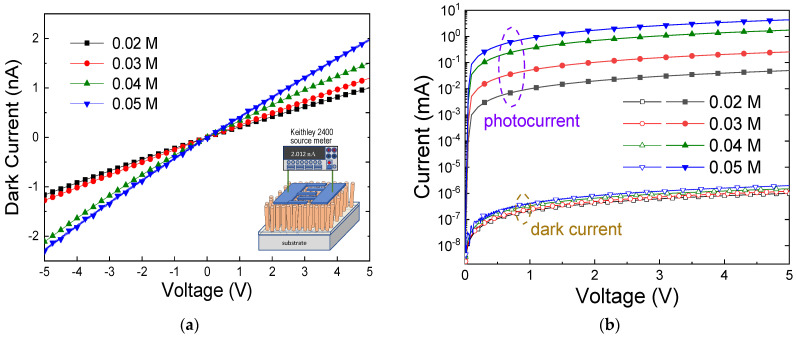
(**a**) Dark current and (**b**) photocurrents of the ZNA-AgNM-based devices prepared with different zinc acetate solution concentrations.

**Figure 11:**
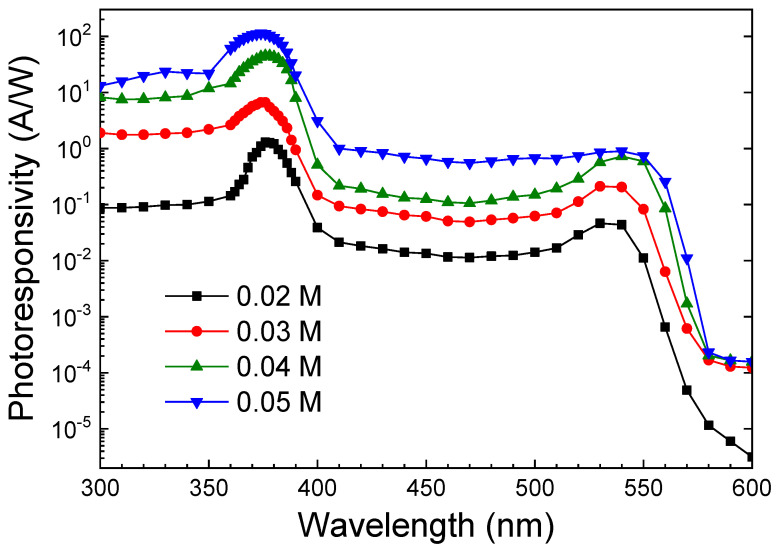
Photoresponsivity of the ZNA-AgNM-based devices as a function of light wavelength at a bias of 5 V.

To evaluate the UV photodetection characteristics of the ZNA-AgNMs, the dark and photocurrents of the MSM devices fabricated with the ZNA-AgNMs were measured. Figure 10a,b display current–voltage (I–V) characteristics of the devices under dark and UV light illumination (λ = 370 nm) for different zinc acetate concentrations. In Figure 10a, the linear I–V curves indicate an ohmic contact between the ZNA and Al electrodes, suggesting minimal resistance at the metal–semiconductor junction, which is crucial for maximizing the sensing response. The dark currents of the devices increased with applied voltage across all concentrations but remained very low, approximately a couple nA within the −5 to 5 V voltage range. When the ZNA is exposed to UV light, photons excite electrons from the valence band to the conduction band, creating holes in the valence band. The increase in hole concentration leads to the chemical desorption of surface-adsorbed oxygen species, which reduces the depletion layer near the ZnO surface and enhances the conductivity of ZnO [10,44]. Both dark and photocurrents increased with higher precursor concentrations, indicating that higher precursor concentrations result in greater ZNA-AgNM conductivity. This enhanced conductivity is likely due to improved crystal quality and larger volume at higher precursor concentrations. The photoresponsivity (*R*) was defined by the following formula [45]:

(12) $$R=\frac{I_{photo}-I_{dark}}{{power}_{in}},$$

where *I_photo_* and *I_dark_* are photocurrent and dark current, respectively, and *power_in_* is the power of incident light. Figure 11 shows photoresponsivity as a function of light wavelength at a bias of 5 V. The photoresponsivity peaked in the UV region, with a notable decrease at 380 nm and 550 nm as the wavelength increased. Table 2 lists the photoresponsivity for the UV, green, and red light bands. The ZNA-AgNM-based photodetector, fabricated with a zinc acetate concentration of 0.05 M, achieved a maximum photoresponsivity of 114 A/W at a wavelength of 374 nm. This exceptionally high photoresponsivity far exceeds that of most commercial UV detectors, which typically range from 0.1 to 0.2 A/W [46].

A comparison of the photodetection performance of the other nanostructured ZnO UV photodetectors related to this study is presented in Table 3. The high photoresponsivity can be attributed to the following mechanisms: (1) When the photon energy exceeds the semiconductor band gap, electron–hole pairs are generated, and these photogenerated charges are extracted to produce photocurrent. (2) The holes in ZnO migrate to the surface along the potential gradient caused by energy band bending, releasing negatively charged adsorbed oxygen ions (h^+^ + O_2_^−^(ad) → O_2_(g)) and photodesorbing oxygen on the surface. Additionally, holes may become trapped on the ZnO surface, increasing the free carrier concentration and reducing the depletion layer width. These effects are particularly significant in nanostructured films, where the large surface area allows the depletion region to extend throughout the film, leading to enhanced carrier injection and transport, which in turn generates a sustained photocurrent [12].

Furthermore, we calculated the UV to green and UV to red rejection ratio, R_uv_/R_green_ and R_uv_/R_red_. The highest R_uv_/R_green_ (203) and R_uv_/R_red_ (7.26 × 10^5^) were obtained at a zinc acetate concentration of 0.05 M. The higher green light response compared to red light is attributed to intrinsic and surface defects such as oxygen vacancies, zinc interstitials, zinc vacancies, and oxygen interstitials, and absorbed hydroxyl group (as discussed in Section 3.2, Figure 7) [26,47]. The high UV–visible rejection ratio indicates that the ZNA-AgNM devices are “visible-blind”. The specific detectivity (D*), another key figure of merit for evaluating the photodetector performance and noise rejection, can be estimated using the following formula [4]:

(13) $$D^{*}=\frac{R}{\sqrt{2qJ_{dark}}},$$

where *q* is the elementary charge, and *J_dark_* is the dark current density. Due to the high photoresponsivity and low dark current, the calculated detectivity of the ZNA-AgNM-based photodetector reached 6.37 × 10^14^ Jones at 5 V as the zinc acetate concentration increased to 0.05 M (see Table 1). This high detectivity indicates that the developed photodetector has strong noise suppression capabilities. The superior performance is attributed to the ZNAs grown at a zinc acetate concentration of 0.05 M, which exhibit better crystallinity and larger volumes per unit area (see Figure 5 and Figure 6), These factors enhance the absorption of UV light by the nanorods and reduce the likelihood of photogenerated carriers being trapped by defects, thereby increasing the photocurrent.

**Table 3:** A comparison of the present study with the literature on the photodetection performance of nanostructured ZnO UV photodetectors.

Device	Wavelength/ Voltage	Responsivity (A/W)	Detectivity (Jones)	Rise/Decay Time	Reference
Ag nanoparticle-ZNA	395 nm/2 V	0.04	-	-/-	[15]
ZNA	360 nm/5 V	0.878	-	10.3 s/17.7 s	[48]
ZNA	390 nm/−5 V	2.19	-	0.49 s/1.14 s	[49]
PEI-ZNA	365 nm/5 V	43.7	1.85 × 10^14^	4.8 s/4.9 s	[44]
ZNA/CuO/p-GaN	365 nm/0 V	1.44 × 10^−3^	5.9 × 10^10^	0.12 s/0.22 s	[50]
ZNA/PEDOT:PSS	364 nm/−2 V	9.2	7.3 × 10^12^	336 s/294 s	[51]
Ag-doped ZNA	365 nm/5 V	0.0173	2.18 × 10^11^	7.4 s/26.9 s	[52]
Ga-doped ZNA	360 nm/1 V	0.046	3.92 × 10^12^	89 s/106 s	[53]
Ga-doped ZnO NWs	365 nm/0 V	0.233	4.18 × 10^12^	159 ms/150 ms	[54]
ZnO NWs on Ag NWs + Ag-paste	365 nm/2 V	54	-	0.2 s/0.1 s	[55]
ZnO/Ag NWs/ZnO	365 nm/1 V	0.1	6.8 × 10^12^	2.15 s/2.44 s	[56]
ZNA on ZnO-AgNM layer	374 nm/5 V	114	6.37 × 10^14^	-/-	This work

## 4. Conclusions

In conclusion, this study successfully demonstrated the hydrothermal growth of ZnO nanorod arrays (ZNA) on silver nanowire mesh (AgNM) substrates using varying zinc acetate concentrations. The morphological, structural, optical, and sensing characteristics of ZNA-AgNM were thoroughly investigated. The AgNM exhibited a densely cross-connected structure with a sheet resistance of 7.23 Ω/□ and a visible light transmittance of approximately 79%. The ZNA structures displayed well-aligned hexagonal nanorods, where the dimensions and areal density varied significantly with changes in zinc acetate concentration. Higher precursor concentrations yielded larger nanorod diameters and volumes but reduced areal density and surface-to-volume ratios. Photodetectors based on ZNA-AgNM showed enhanced conductivity with increasing zinc acetate concentration, evidenced by increased dark current and photocurrent. Notably, at a zinc acetate concentration of 0.05 M, the photodetectors achieved a maximum photoresponsivity of 114 A/W at 374 nm, with UV-to-green and UV-to-red rejection ratios of 2.03 × 10^2^ and 7.26 × 10^5^, respectively, and a specific detectivity of 6.37 × 10^14^ Jones, indicating optimized noise suppression and UV detection capabilities. Furthermore, the hydrogen sensing capabilities of ZNA-AgNM-based gas sensors were explored, revealing increased response with rising measurement temperatures, peaking at 2.71 at 300 °C/2000 ppm for sensors prepared with 0.04 M zinc acetate concentration. This study underscores the effectiveness of zinc acetate precursor concentration in tuning the structural, optical, and sensing properties of ZNA-AgNM. These findings highlight the potential of ZNA-AgNM for high-performance UV photodetectors and hydrogen sensors in the future.

## Figures and Tables

**Table 1:** A comparison of gas sensing performance of the ZnO-based H_2_ gas sensor with previous works.

Device	H_2_ Concentration (ppm)	Operating Temperature	Response (Sensitivity)	Response/Recovery Time	Reference
ZNA	500	180 °C	|I_a_ − I_g_|/I_a_ = 0.26	-/-	[14]
ZnO with a MoS_2_ nanosheet catalyst	100	250 °C	R_a_/R_g_~4	7 s/23 s	[30]
single ZnO NW	100	RT	|R_a_ − R_g_|/R = 0.34	64 s/11 s	[34]
Pd nanoparticles-ZNA	10,000	RT	(V_a_ − V_g_)/V_g_ = 0.373	100 s/-	[35]
1-D ZnO NWs	250	300 °C	|G_a_ − G_g_|/G_g_ = 1.66	>300 s/>300 s	[38]
Ag-doped ZnO thin films	1000	300 °C	|I_a_ − I_g_|/I_a_ = 0.46	>300 s/>300 s	[39]
Ag/Pd nanoparticle-ZnO nanoplates	500	400 °C	R_a_/R_g_ = 78	2 s/13 s	[40]
Pt nanoparticles-ZnO pencil-like microstructures	100	150 °C	|R_a_ − R_g_|/R_a_ = 0.638	193 s/378 s	[41]
Pd–ZnO nanosheets	50	250 °C	R_a_/R_g_ = 2.51	336 s/294 s	[42]
ZnO nanostructured thin film	1200	400 °C	(R_a_ − R_g_)/R_a_ = 0.23	110 s/-	[43]
ZNA on ZnO-AgNM layer	2000	300 °C	R_a_/R_g_ = 2.71 (R_a_ − R_g_)/R_a_ = 0.631	100 s/96 s	This work

**Table 2:** Photoresponsivity, rejection ratio, and specific detectivity at different precursor concentrations under a bias of 5 V.

Concentration (M)	0.02	0.03	0.04	0.05
R_UV_ (A/W)	1.29	6.95	4.53 × 10^1^	1.14 × 10^2^
R_green_ (A/W)	8.60 × 10^−2^	6.30 × 10^−2^	4.50 × 10^−1^	5.62 × 10^−1^
R_red_ (A/W)	3.15 × 10^−6^	1.23 × 10^−4^	1.57 × 10^−4^	1.57 × 10^−4^
R_UV_/R_green_	1.50 × 10^2^	1.10 × 10^2^	1.01 × 10^2^	2.03 × 10^2^
R_UV_/R_red_	4.10 × 10^5^	5.65 × 10^4^	2.89 × 10^5^	7.26 × 10^5^
Detectivity (Jones)	1.02 × 10^13^	5.02 × 10^13^	2.92 × 10^14^	6.37 × 10^14^

## Data Availability

Data are contained within the article.
